# The Role of ATP-Binding Cassette Proteins in Stem Cell Pluripotency

**DOI:** 10.3390/biomedicines11071868

**Published:** 2023-06-30

**Authors:** Prince Saini, Sharath Anugula, Yick W. Fong

**Affiliations:** 1 Brigham Regenerative Medicine Center, Brigham and Women’s Hospital, Boston, MA 02115, USA; psaini@bwh.harvard.edu (P.S.); sanugula@bwh.harvard.edu (S.A.); 2 Department of Medicine, Cardiovascular Medicine Division, Harvard Medical School, Boston, MA 02115, USA; 3 Harvard Stem Cell Institute, Cambridge, MA 02138, USA

**Keywords:** ABC transporters, pluripotency, cell signaling, metabolism, phospholipids, glutathione, reactive oxygen species

## Abstract

Pluripotent stem cells (PSCs) are highly proliferative cells that can self-renew indefinitely in vitro. Upon receiving appropriate signals, PSCs undergo differentiation and can generate every cell type in the body. These unique properties of PSCs require specific gene expression patterns that define stem cell identity and dynamic regulation of intracellular metabolism to support cell growth and cell fate transitions. PSCs are prone to DNA damage due to elevated replicative and transcriptional stress. Therefore, mechanisms to prevent deleterious mutations in PSCs that compromise stem cell function or increase the risk of tumor formation from becoming amplified and propagated to progenitor cells are essential for embryonic development and for using PSCs including induced PSCs (iPSCs) as a cell source for regenerative medicine. In this review, we discuss the role of the ATP-binding cassette (ABC) superfamily in maintaining PSC homeostasis, and propose how their activities can influence cellular signaling and stem cell fate decisions. Finally, we highlight recent discoveries that not all ABC family members perform only canonical metabolite and peptide transport functions in PSCs; rather, they can participate in diverse cellular processes from genome surveillance to gene transcription and mRNA translation, which are likely to maintain the pristine state of PSCs.

## 1. Introduction

Pluripotent stem cells (PSCs) are derived from the inner cell mass of the blastocyst [1,2]. Expansion of these cells during embryonic development or in vitro through self-renewal requires coordinated changes in cellular metabolism [3]. Like other rapidly dividing cells, PSCs must amplify their macromolecular contents such as nucleic acids, carbohydrates, proteins, lipopolysaccharides, and lipids, by generating precursor molecules to meet the metabolic requirements of cell proliferation. However, increasing evidence indicates that cellular metabolism not only plays an important role in regulating proliferative capacity but also self-renewal versus the differentiation of PSCs [4,5]. For example, lipid metabolites can act as signaling molecules and activate signaling pathways that converge on a unique network of genes controlled by stem cell-enriched transcription factors OCT4 and SOX2 [6]. OCT4 and SOX2 co-regulate a large number of genes to sustain stem cell pluripotency [7,8,9,10]. Intrinsic and extrinsic signals that perturb this transcriptional network impair PSC self-renewal and promote differentiation [11]. Therefore, regulating the availability and distribution of lipids and other macromolecules in PSCs could modulate cell signaling and influence the cell fate decision. Metabolite homeostasis reflects a balance between synthesis and degradation or export [12]. While the biosynthetic pathways of essential macromolecules are well-understood, the role of transporters in regulating their concentration and distribution remains underexplored.

The plasticity of PSCs is in part facilitated by the prevalence of open chromatin and elevated global transcriptional activities [13]. However, the permissive chromatin structure and the act of transcription itself are sources of genome instability, exposing DNA to DNA-modifying enzymes and genotoxic agents such as reactive oxygen species (ROS) [14,15]. In addition, unlike somatic cells, PSCs display a “compressed” cell cycle with a shortened G1, resulting in increased replicative stress [16]. Furthermore, PSCs do not undergo DNA damage-induced G1 cell cycle arrest, which in somatic cells is thought toprovide time to repair critical damage before DNA replication occurs [17,18]. Therefore, ESCs are presumably at higher risk of acquiring mutations. Paradoxically, it has been found that the apparent mutation frequency in PSCs is about 100-fold lower than that in somatic cells [19], suggesting that there are additional mechanisms in PSCs that suppress mutagenesis and/or purge damaged cells. PSCs respond to DNA damage by undergoing rapid differentiation and apoptosis [20,21]. It is thought that hypersensitivity to DNA damage prevents deleterious mutations in PSCs from becoming amplified and propagated to progenitor cells [22]. Therefore, a regulated transcriptional switch from self-renewal to differentiation is not only important for embryonic development but also for genome maintenance in PSCs.

The seminal discovery that the PSC fate can be induced in somatic cells via the ectopic expression of a cadre of transcription factors opens the possibility of generating patient-specific induced pluripotent stem cells (iPSCs) for regenerative medicine [23,24]. While iPSCs are highly similar to bona fide ESCs [25], studies indicated that iPSC lines display altered gene expression patterns [26] and recurrent genetic abnormalities [26,27,28] that have been shown to increase the risk of tumorigenicity [29,30,31], thus posing serious challenges to using iPSCs for regenerative medicine due to significant safety concerns [31,32,33,34]. In order to fully realize the therapeutic potential of iPSCs, we suggest that a more complete understanding of the molecular underpinnings of stem cell pluripotency is required.

In this review, we focus on the “canonical” roles of membrane-bound ATP-binding cassette (ABC) transporters in the translocation of lipids, cholesterol, and ROS-scavenging glutathione peptides in PSCs, and the implications for modulating cellular signaling and homeostasis critical for stem cell pluripotency. We also discuss the unexpectedly diverse functions of non-membrane-bound ABC proteins in translation and in coordinating stem cell-specific transcription with genome surveillance, to maintain a pristine proteome and genome for proper stem cell function and regenerative medicine.

## 2. ABC Expression in PSCs

**Figure 1:**
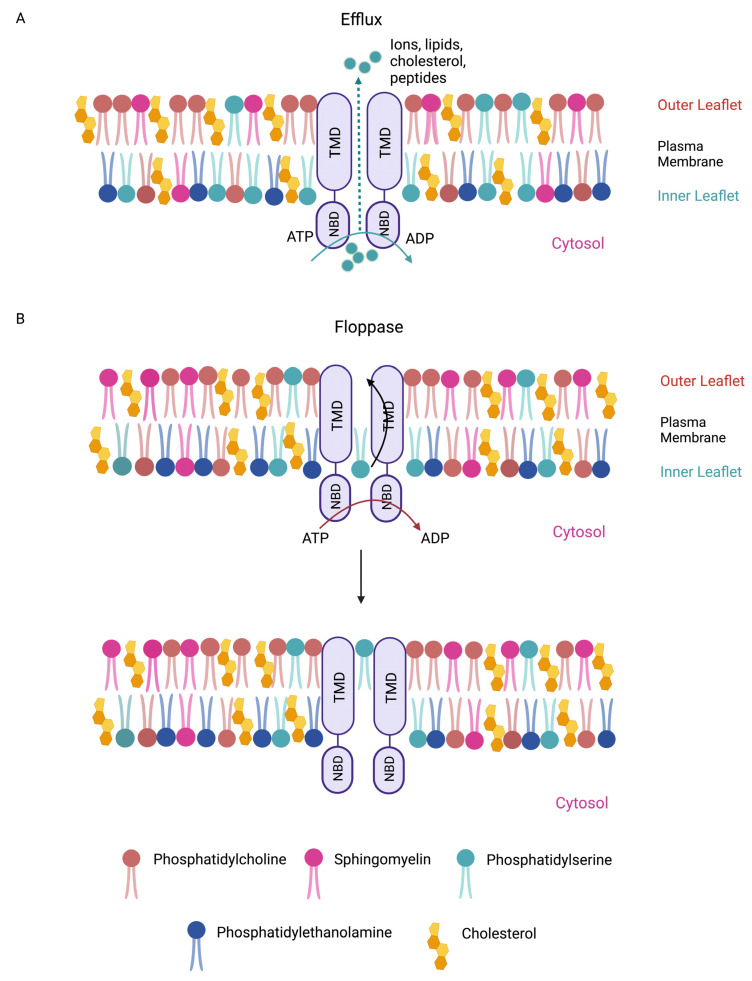
Membrane-bound mammalian ABC transporters are efflux pumps and/or floppases. (**A**) ABC proteins are essential membrane-bound transporters. ABC transporters are anchored at cell membranes through their transmembrane domains (TMDs). ABC transporters can efflux ions and macromolecules (e.g., lipids, cholesterol, and peptides) across cell membranes. (**B**) ABC transporters are also lipid floppases. They are critical for maintaining the asymmetric distribution of phospholipids in the membrane. Phosphatidylcholine (beige) and sphingomyelin (pink) reside predominantly in the outer leaflet of the plasma membrane, whereas anionic lipids such as phosphatidylserine (cyan) and phosphatidylethanolamine (blue) are more prevalent in the inner leaflet. Membrane cholesterols are depicted (yellow). Phospholipids such as phosphatidylserine from the inner membrane leaflet are “flopped” to the outer leaflet. Efflux and floppase activities require ATP hydrolysis by the nucleotide-binding domains (NBDs).

Ubiquitous from bacteria to humans, the ABC superfamily is one of the largest classes of transmembrane (TM) proteins [35]. In mammals, membrane-bound ABC proteins are efflux transporters that translocate essential substrates ranging from ions to macromolecules across membranes at the cost of ATP hydrolysis (Figure 1). It is perhaps not surprising that defects in these transporters are associated with human disorders, including metabolic diseases (Table 1) [36,37]. Of the 49 *ABC* genes in the human genome, 4 lack TM domains and thus are not transporters [38]. Transcriptomic and proteomic studies indicated that 26 *ABC* genes are expressed in PSCs [39,40,41,42] (Table 1), and that their expression levels change when PSCs exit from pluripotency and undergo differentiation [39,42,43]. However, the lack of specific antibodies against some of these ABC proteins precludes a confirmation of their expression at the protein level. Nonetheless, drawing on observations in PSCs and other cell systems, we suggest that cell type-specific expression patterns of ABC proteins not only reflect differences in metabolic requirements but may also contribute to cell fate regulation.

## 3. The Roles of ABC Transporters in PSCs

### 3.1. Lipid Transporters (ABCA1 and ABCC1)

Lipids are a diverse class of biomolecules. Glycerophospholipids, specifically phosphatidylcholine (PC), phosphatidylethanolamine (PE), phosphatidylserine (PS), and phosphatidylinositol (PI), as well as sphingolipids and cholesterol, serve as building blocks for membranes and organelles [51]. Some ABC transporters (ABCC1 [52,53]) act as “floppases” by catalyzing the movement of specific phospholipid species from the cytosolic leaflet to the extracellular leaflet of the plasma membrane (PM) [54], while others (ABCA1 [55]) function as extracellular phospholipid translocases (Figure 1). Indeed, ABC transporters have been shown to contribute to the asymmetric distribution of different phospholipids in the lipid bilayer, with PC and sphingolipids such as sphingomyelin (SM) residing predominantly in the outer leaflet of the PM, whereas anionic lipids such as PE, PS, and PI accumulate in the inner leaflet [56,57]. Increasing evidence indicates that changes in the composition and distribution of these phospholipids in the lipid bilayer can regulate signal transduction pathways that are known to regulate PSC cell fates [58,59]. 

Stem cell maintenance in human PSCs requires basic fibroblast growth factor (bFGF), which activates the RAS-RAF-MEK-ERK signal transduction cascade [60,61,62,63]. The association of RAS with the inner leaflet of the PM is an important step in the recruitment and activation of its effectors such as RAF and phosphatidylinositol 3-kinase (PI3K) [64]. Interestingly, it has been shown that RAS can adopt a distinct orientation at the PM, depending on the types of phospholipids (PC, PS, or phosphatidylinositol 4,5-bisphosphate [PIP2]) that interact with RAS [65]. As a result, the catalytic domain of membrane-bound RAS is predicted to become more exposed or partially obscured. Therefore, how RAS is anchored in the PM could modulate its ability to interact with its effectors (e.g., RAF versus PI3K) and regulate RAS-mediated downstream signaling choices. It appears that electrostatic interactions between RAS and lipids dictate interaction affinity and orientation preferences. Given that ABC transporters can translocate PC, PS, and PIP2 to the cell membrane outer leaflet [66,67,68], we propose that changes in the local distribution of phospholipids in the lipid bilayer by specific ABC transporters could influence the spatial arrangement of RAS. Future studies will be required to address the expression patterns of ABC transporters and their function in regulating the distribution of membrane phospholipids and RAS signal transduction, thereby controlling stem cell self-renewal versus differentiation. In a similar manner, it will be prudent to examine whether or not other signaling pathways (e.g., TGF-β [69] and EGFR [70]) that are known to contribute to stem cell pluripotency can also be modulated by PM phospholipid organization. 

### 3.2. Cholesterol Transporters (ABCA1 and ABCG1)

Cholesterol is an important constituent of cell membranes. The bulk of cellular cholesterol (~90%) is localized at the PM [71]. Cholesterol homeostasis is determined by the biosynthesis, uptake, and efflux of cholesterol. ABCA1 and ABCG1 play crucial roles in the efflux of cellular cholesterol and thus are important regulators of membrane cholesterol level [72,73,74].

Cholesterol is a key modulator of membrane fluidity [75,76], which in turn regulates cell behaviors such as adhesion, proliferation, and migration [77]. However, recent evidence indicates that changes in PM stiffness may also regulate cell fate changes in PSCs [78]. It has been shown that the rigidification of the PM precedes or coincides with downregulation of gene expression programs that stabilize the pluripotent state in PSCs, suggesting that a decrease in membrane fluidity may prime PSCs to exit from pluripotency. Consistent with the notion that maintenance of membrane fluidity contributes to stem cell maintenance, enzymes in the cholesterol biosynthesis pathways have been shown to be expressed at higher levels in PSCs, thereby increasing membrane cholesterol content and fluidity [78,79]. Importantly, the inhibition of cholesterol production in PSCs accelerates their exit from pluripotency, as indicated by the rapid downregulation of stem cell marker alkaline phosphatase [78]. These observations underscore the importance of cholesterol homeostasis in stem cell maintenance. We propose that dissecting the mechanisms by which the expression and activities of ABCA1 and ABCG1 are controlled in PSCs will advance our understanding of the role of cholesterol efflux in regulating membrane fluidity and stem cell pluripotency. 

In addition to regulating membrane fluidity, cholesterol, together with SM, has been shown to assemble dynamic, cholesterol-rich microdomains in the outer leaflet of the PM [80]. These compartmentalized domains, known as lipid rafts, have been shown to enrich specific receptors and their effectors to promote receptor–effector interactions, thereby lowering activation barriers. The ability of lipid rafts to partition and concentrate select signaling machineries depends on the intrinsic affinity of these signaling proteins to lipid rafts, which has been shown to be influenced by amino acid sequences in the TM domains of membrane receptors and protein palmitoylation [81,82]. Oligomerization of receptors has also been reported to increase their affinity to lipid rafts and residence time in these lipid subdomains [83], hinting at a potential mechanism by which lipid rafts amplify signaling. We suggest that a small change in the concentration of signaling components in lipid rafts may be sufficient, through amplification, to initiate signaling cascades. Therefore, lipid rafts may play an important role in increasing the responsiveness of signal transduction machineries to cellular stimuli.

It has been shown that ABCA1 and ABCG1 deficiency in macrophages leads to an increase in the number of lipid rafts and enhanced signaling responses [84]. This is likely due to the propensity of lipid rafts to cluster, resulting in the amplification of signals [85,86]. These observations suggest an inhibitory function of ABCA1 and ABCG1 in lipid raft formation, via the mobilization of cholesterol from lipid rafts to non-raft domains. It will be of interest to determine the mechanisms by which ABC transporters are recruited to lipid rafts. This is because the active efflux of membrane cholesterol by ABC transporters could facilitate the fine-tuning and dissolution of signal transduction hubs in lipid rafts and signal termination.

Lipid rafts are also detected in PSCs, but their roles in stem cell maintenance are less well-understood [87]. The self-renewal of mouse PSCs requires leukemia inhibitory factor (LIF) signaling [88]. It has been shown that depletion of membrane cholesterol in mouse PSCs by methyl-β-cyclodextrin (Mβ-CD), which has been shown to disrupt lipid rafts, compromises the recruitment of LIF receptor and its co-receptor gp130 to rafts and blunts LIF receptor-JAK-STAT3 signaling [87]. The observed reduction in expression levels of key pluripotency-associated transcription factors OCT4 and SOX2 in Mβ-CD-treated PSCs indicates a destabilized pluripotent state when lipid raft formation is impaired. These observations are consistent with the role of lipid rafts in enriching specific receptors and facilitating their activation. Lipid rafts have also been implicated in other signaling pathways that are known to promote stem cell self-renewal and pluripotency, such as EGFR [70] and RAS [89], and those that destabilize the stem cell state, including insulin receptor [90] and hedgehog [91]. An outstanding question is how ABC transporters may control lipid raft formation and dynamics to partition competing signaling in PSCs to favor self-renewal over differentiation. 

### 3.3. Redox Regulation and Oxidative Stress (ABCC1 and ABCC4)

ROS are natural byproducts of cellular metabolism. ROS can cause damage to the basic building blocks of cells including DNA, protein, and lipids. Therefore, ROS pose significant threats to the ability of PSCs to maintain genome and proteome integrity as they self-renew. In addition to cellular damages inflicted by ROS build-up, an imbalance in ROS levels can also lead to the misregulation of redox sensor molecules via the oxidation of cysteine residues. Some of these redox sensors are key signaling effectors such as AKT and MAPK [92,93]. Therefore, it is conceivable that an increase in ROS concentration destabilizes the pluripotent cell state in part by interfering with signaling pathways essential for stem cell maintenance [94]. ROS levels in cells are determined by the rate of ROS generation and the rate of ROS scavenging by antioxidants. PSCs are able to maintain relatively low ROS levels compared to those of differentiated cells, in part due to their reliance on glycolysis rather than oxidative phosphorylation for energy production, which is known to generate less ROS [95,96]. Nevertheless, the neutralization of ROS species by antioxidants remains a critical mechanism in regulating ROS homeostasis in PSCs as it is essential for stem cell maintenance [97,98].

Glutathione (GSH) is a major antioxidant in cells [99,100]. GSH levels are balanced by its synthesis, transport, efflux, and degradation. Studies have shown that ABCC1 is a major GSH exporter and can regulate intracellular GSH levels. The overexpression of ABCC1 reduces intracellular GSH levels, while ABCC1 deficiency increases GSH concentrations [101,102]. Importantly, ABCC1 can export both GSH and various oxidized glutathione derivatives (e.g., glutathione disulfide (GSSG)), although with distinct substrate affinity [103,104,105]. Therefore, in addition to cellular enzymes that can degrade GSH (e.g., CHAC1 [106,107]) or regenerate GSH from GSSG (e.g., GSH reductase [108]), ABCC1 likely plays an integral role in maintaining the redox equilibrium in PSCs. It has been shown that oxidative stress downregulates key PSC-specific transcription factors OCT4 and SOX2, and compromises AKT signaling [97]. While the precise mechanism is unclear, the destabilization of OCT4 proteins and inactivation of AKT via the oxidation of critical cysteines residues could compromise the gene transcription and cellular signaling required for stem cell maintenance [92,109,110]. 

Like oxidative stress, reductive stress induced by excessive levels of GSH can also impair PSC functions. Physiological levels of ROS have been shown to promote PSC proliferation and accurate DNA synthesis [111]. High concentrations of antioxidants interfere with cell cycle progression and lead to the accumulation of DNA breaks [112], likely due to the toxic effects of high antioxidant levels on the stability of cell cycle regulators and proteins involved in the DNA damage response and DNA repair [111]. The balance between ROS and antioxidants must be optimal, as both extremes, oxidative and reductive stress, are damaging to PSCs. Functional studies on the role of ABCC1 and ABCC4 in PSCs will address the precise role of GSH/GSSG efflux in establishing a cellular redox state favorable for stem cell self-renewal and genome maintenance.

## 4. Non-Canonical Functions of ABCs in PSCs

While most ABC proteins are membrane-bound transporters, ABCE1 and the ABCF subfamily proteins (ABCF1, ABCF2, and ABCF3) lack TM domains [38]. Although their precise functions remain somewhat enigmatic, recent studies highlight the multifaceted function of ABCF1 in regulating translation, innate immune response, and transcription, thus expanding the functional repertoire of ABC proteins. 

### 4.1. ABCF1 in mRNA Translation

**Figure 2:**
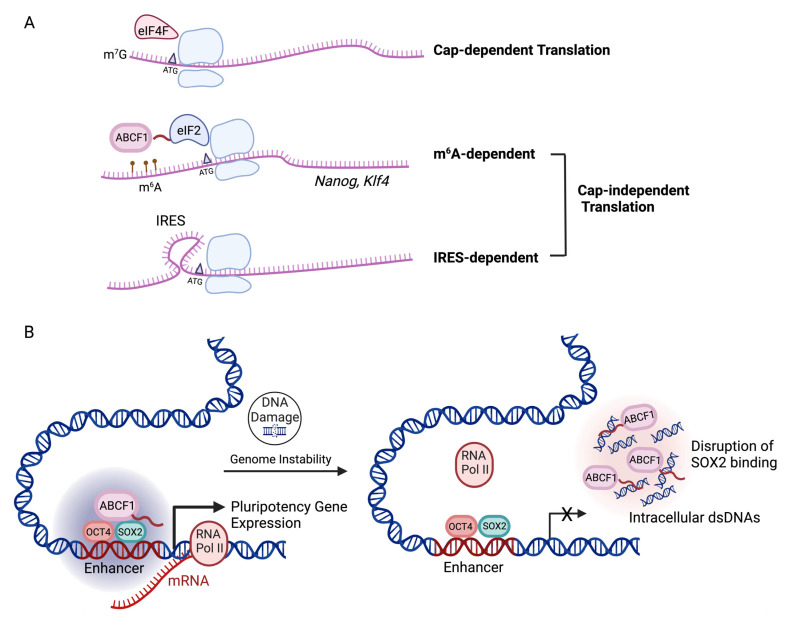
Multifaceted roles of ABCF1 in translation and transcription in PSCs. ABCF1 lacks TMD but contains a low-complexity domain (LCD) in the N-terminus critical for ABCF1 functions in PSCs. (**A**) Diagram showing that cap-dependent translation of m^7^G capped mRNAs requires binding of translation initiating factor eIF4 to the cap structure. ABCF1 may promote the cap-independent translation of m^6^A-modified pluripotency-associated mRNAs (e.g., *Nanog* and *Klf4*) in mouse PSCs, via an interaction with eIF2 through its LCD. Internal ribosome entry site (IRES)-mediated cap-independent translation is not dependent on ABCF1. (**B**) A diagram showing that ABCF1 acts as transcriptional coactivator for SOX2 in PSCs. The LCD in ABCF1 directly interacts with SOX2 and assembles transcriptional complexes at pluripotency gene enhancers essential for gene activation. Upon DNA damage in PSCs, the LCD-dependent interaction of ABCF1 with SOX2 is disrupted due to competitive binding between ABCF1 and aberrant intracellular DNAs that accumulate in damaged PSCs. This leads to the downregulation of pluripotency gene expression and exit of damaged PSCs from self-renewal.

The initiation of mRNA translation can occur via cap-dependent and independent mechanisms [113,114]. In addition to internal ribosome entry site (IRES) elements in mRNAs, RNA methylation at adenosines (m^6^A) by m^6^A methyltransferases such as METTL3 has been shown to also facilitate cap-independent translation initiation [115,116]. m^6^A is the most abundant modification on mRNAs [117]. m^6^A modifications have been shown to influence mRNA splicing and nuclear export [118], and regulate mRNA stability by targeting transcripts for degradation in RNA decay bodies [119,120]. In PSCs, mRNAs encoding core pluripotency transcription factors such as *Nanog* and *Klf4* are also marked by m^6^As. However, it is less clear how PSCs overcome the destabilization effect of m^6^A modification on core pluripotency gene transcripts to ensure their robust expression, which is necessary for self-renewal. A recent study suggested a potential active mechanism to translate m^6^A-modified mRNAs [121]. It has been shown that ABCF1 promotes the cap-independent translation of m^6^A-modified mRNAs, likely by facilitating the recruitment of the eukaryotic initiation factor 2 (eIF2) ternary complex in the absence of cap recognition machinery (Figure 2A). We speculate that ABCF1 may function to stabilize the pluripotent state during cellular stress, by ensuring the efficient translation of these pluripotency-associated transcripts when global cap-dependent translation is inhibited [122]. 

ABCF1 displays some sequence similarity to the yeast eEF3 subfamily ABC proteins including general control non-derepressible-20 (GCN20), which have been implicated in translational control [123,124]. However, the homology is restricted to the nucleotide-binding domains (NBDs). The residues outside of NBDs in ABCF1 are highly divergent from GCN20. Nonetheless, studies have shown that both ABCF1 and GCN20 employ their unique N-terminal regions to interact with eIF2 [124,125]. While the reported ABCF1-dependent translation has only been studied in mouse embryonic fibroblasts (MEFs), it is likely that this mechanism is also conserved in PSCs. Because ABCF1 expression is significantly higher in PSCs compared to that in somatic cells [48,126], we surmise that ABCF1 may play a more prominent role in the efficient translation of m^6^A-modified mRNAs critical for stem cell pluripotency. 

### 4.2. ABCF1 as an Intracellular DNA Sensor

Studies on differentiated mouse cells allowed researchers to identify ABCF1 as a sensor for aberrant intracellular DNAs [127,128]. ABCF1 interacts with critical regulators of the innate immune response and activates a pro-inflammatory response to intracellular DNAs resulting from infection or DNA damage [103,127], thereby promoting apoptosis and clearance of the affected cells [129]. While PSCs express ABCF1 and other known DNA sensors (e.g., cGAS and STING [130]), downstream signaling pathways required to stimulate the production of pro-inflammatory cytokines are absent or highly attenuated, in part due to active suppression by stem cell-specific transcription factors including OCT4 and SOX2 [131,132]. Whether or not ABCF1 also recognizes intracellular DNAs in PSCs and the biological consequences is unknown. Our recent work indicates that PSCs co-opt ABCF1′s ability to detect intracellular DNAs to modulate stem cell-specific transcription in response to genome instability (discussed in the next sections) [48,133].

### 4.3. ABCF1 as a Stem Cell-Specific Transcriptional Coactivator

The unique transcriptional signatures that define the PSC state require cooperation between PSC-specific transcription factors and their coactivators [134,135]. Transcription factors OCT4 and SOX2 co-regulate a large number of genes that determine whether or not PSCs undergo self-renewal as they expand in the inner cell mass or commit to differentiation during embryonic development [7,8,136]. Therefore, the transcriptional activities of OCT4 and SOX2 are tightly regulated. Previous studies implicated the MED1 subunit of cell-ubiquitous coactivator complex Mediator in regulating OCT4 activity via a direct interaction [137,138]. However, other studies suggested the requirement of PSC-specific coactivators [139]. To this end, our laboratory developed an in vitro transcription assay and in an unbiased manner screened for factors in PSC nuclear extracts that can stimulate transcriptional activation by OCT4 and SOX2 [140]. We identified ABCF1 as a critical coactivator for OCT4 and SOX2 in PSCs. ABCF1 contains an unusual N-terminal region that is composed primarily of lysine and glutamic acid residues (40%). ABCF1 potentiates transcription by utilizing this low-complexity sequence domain (LCD) to interact directly with SOX2 and assemble PSC-specific transcriptional complexes at pluripotency-associated gene promoters (Figure 2B). Importantly, the yeast homologue GCN20 cannot be substituted for ABCF1 in transcriptional activation because the N-terminal region in GCN20 is highly divergent from the LCD of ABCF1. These observations suggest the acquisition of a mammalian-specific function of ABCF1 in transcriptional control. *Abcf1* knockout mouse embryos die at 3.5 days post coitus, a developmental stage that coincides with the emergence of pluripotent cells in the inner cell mass of the blastocyst [126]. Thus, genetic evidence indicates that ABCF1 is an essential transcriptional regulator of stem cell pluripotency.

The structural flexibility of the LCD in ABCF1 likely allows the rapid remodeling of transcriptional complexes to induce dynamic changes in gene expression to regulate stem cell self-renewal versus differentiation. LCDs are prevalent in transactivation domains in transcription factors [141]. The unique ability of LCDs to establish transient and multivalent interactions has been shown to allow transcription factors and coactivators to coalesce and overcome activation barriers [142]. The flexible nature of LCDs is also thought to facilitate the dynamic interaction with multiple protein partners, by virtue of their ability to rapidly adopt an ensemble of conformations [143].

### 4.4. ABCF1 Couples Transcription and Genome Surveillance in PSCs

PSCs appear to have developed several mechanisms to reduce the mutational load caused by elevated replicative and transcriptional stress [22]. As discussed in Section 1, damaged PSCs are efficiently eliminated through enforced exit from self-renewal via differentiation, thereby preserving the genome integrity of the self-renewing PSC population. DNA damage-induced PSC differentiation first requires the dismantling of the pluripotency gene transcriptional network that supports self-renewal, followed by the activation of differentiation programs. The tumor suppressor p53 has been proposed to regulate this transcriptional switch [144,145]. However, other studies indicated that the downregulation of the pluripotency gene network still occurs in ESCs lacking p53 [20,146]. The global shutdown of transcription upon DNA damage also cannot fully account for the transcriptional switch observed in damaged ESCs [147,148,149]. These observations suggest additional regulators that can relay signals from DNA damage to selectively modulate pluripotency gene transcription.

Our recent studies on the transcriptional function of ABCF1 revealed a new link between transcription and genome surveillance in PSCs [48]. Upon DNA damage, we found that ABCF1 binds intracellular DNAs that accumulate in damaged PSCs at the expense of its interaction with SOX2 (Figure 2B). The observed competition is likely due to the fact that both SOX2 and DNAs compete for the same LCD for binding. The disruption of an ABCF1-SOX2 complex by intracellular DNAs results in the dissociation of ABCF1 from its target pluripotency gene promoters, the downregulation of pluripotency gene expression, and differentiation of compromised PSCs. While DNA sensing by ABCF1 does not activate a canonical innate immune response in PSCs, PSCs appear to take advantage of ABCF1′s intrinsic affinity to intracellular DNAs to modulate ABCF1-SOX2 interactions in the nucleus. We propose that the ABCF1–SOX2 complex represents an important regulatory nexus, wherein the constant tug of war between transcriptional activation and intracellular DNA sensing by ABCF1 could drive a PSC to self-renew under steady-state conditions, or alternatively to commit to differentiation and apoptosis when genome integrity is compromised. This switching of cell fates critically depends on whether or not intracellular DNA rises above a certain threshold that irreversibly tilts the balance toward the rapid exit of pluripotency.

## 5. Conclusions and Perspective

Although changes in metabolism have traditionally been viewed as a byproduct of cell fate changes and growth demands, there is growing evidence that metabolic regulation drives stem cell fate decisions. We have presented in this review evidence that the ABC family proteins contribute to pluripotent cell fate by coordinating an interconnected network of biological processes, from metabolism and signaling cascades involving macromolecule interactions at the cell membrane, to gene transcription and translation. In order for PSCs to dynamically respond to changing cellular cues, activities of ABC proteins must be coordinated and tuned. In this regard, stem cell-enriched transcription factors have been shown to bind the promoters of several *ABC* genes as discussed in this review, suggesting that their expression could be coupled to the pluripotent cell state [150]. Furthermore, activities of ABC transporters can be regulated by protein–protein interactions and post-translational modifications such as phosphorylation [151]. It is noteworthy that the efficient reprogramming of somatic cells to pluripotency also requires ABCF1, lipid and cholesterol metabolism, and an optimal redox status [48,152,153,154]. It is worth noting that the precise role of ABC proteins in stem cell pluripotency remains unclear, in large part because they have not been rigorously profiled and studied in PSCs. In this review, we synthesized observations from non-PSC types and proposed how cellular pathways controlled by ABC proteins may also contribute to stem cell maintenance. Future efforts on unraveling the biological impacts of ABC proteins on cell fate regulation in PSCs will be required. The knowledge gained is expected to significantly impact our understanding of embryonic development and the ability to manipulate PSCs for regenerative medicine.

## Figures and Tables

**Table 1:** Summary of Human ABC proteins and their functions, involvement in diseases and expression in pluripotent stem cells. Abbreviations: PC, phosphatidylcholine; PS, phosphatidylserine; SM, Sphingomyelin; HDL, high-density lipoprotein.

Symbol	Alias	Subcellular Location	Function	Disease Associated	Expression at mRNA/Protein Level in PSCs
ABCA1	ABC1	Plasma membrane, endoplasmic reticulum	Cholesterol efflux onto HDL/phospholipids	Tangier disease	mRNA [39], Protein [43]
ABCA2	ABC2	Endosome, lysosome	Cholesterol, drug resistance	Alzheimer’s disease	mRNA [39]
ABCA3	ABC3	Endosome, lysosome	Surfactant secretion	Surfactant metabolism dysfunction 3	mRNA [39]
ABCA5		Plasma membrane	Cholesterol efflux transporter		mRNA [39]
ABCA7		Plasma membrane, endoplasmic reticulum	Transport PC, PS, and SM from the cytoplasmic to the exocytoplasmic side of membranes,	Alzheimer’s disease	mRNA [39]
ABCB1	PGY1, MDR	Plasma membrane	Glucosylceramides, multidrug resistance	Inflammatory bowel disease	mRNA [44]
ABCB2	TAP1	Endoplasmic reticulum	Peptide transport	Bare lymphocyte syndrome type I	mRNA [39]
ABCB3	TAP2	Endoplasmic reticulum	Peptide transport	Bare lymphocyte syndrome, type I due to TAP2 deficiency	mRNA [39]
ABCB4	PGY3	Plasma membrane	PC transport	Cholestasis 3 (PFIC3)	mRNA [45]
ABCB6	MTABC3	Plasma membrane, endosome, endoplasmic reticulum, Golgi, mitochondria, lysosome	Iron transport/heavy metal importer subfamily and role in porphyrin transport	Dyschromatosis universalis hereditaria 3, Lan blood group	mRNA [39,40], Protein [40,43]
ABCB7	ABC7	Mitochondria	Fe/S cluster transport	X-linked sideroblastic anemia with ataxia	mRNA [39,40,45], Protein [41]
ABCB8	MABC1	Mitochondria	Mitochondrial iron export; organic and inorganic molecules out of the mitochondria		mRNA [39], Protein [40]
ABCB9		Lysosome	ATP-dependent low-affinity peptide transporter		mRNA [39]
ABCB10	MTABC2	Mitochondria	Enhances heme biosynthesis in developing red blood cells		mRNA [39,45,46]
ABCC1	MRP1	Plasma membrane, lysosome	Glutathione and other organic anions, drug resistance		mRNA [39,47], Protein [41,43]
ABCC4	MRP4	Plasma membrane	Cyclic nucleotides, bile acids, and eicosanoids/nucleoside transport/ glutathione		mRNA [39,40], Protein [40]
ABCC5	MRP5	Plasma membrane, endosome Golgi,	Nucleoside transport/glutamate conjugate and analog transporter/cAMP and cGMP, folic acid and N-lactoyl-amino acids		mRNA [39,40]
ABCC10	MRP7	Plasma membrane	Transport of glucuronide conjugates such as estradiol-17-beta-o-glucuronide and GSH conjugates such as leukotriene C4		mRNA [39]
ABCD1	ALD	Peroxisome	Peroxisomal transport of very long fatty acid/adrenoleukodystrophy	X-linked adrenoleuko-dystrophy	mRNA [39], Protein [40]
ABCD3	PXMP1, PMP70	Peroxisome	Peroxisomal transport of very long fatty acid/long-chain fatty acids (LCFA)-CoA, dicarboxylic acids-CoA, long-branched-chain fatty acids-CoA and bile acids from the cytosol to the peroxisome lumen for beta-oxidation		mRNA [39,40,45], Protein [41]
ABCD4	PMP69, P70R	Peroxisome, lysosome, endoplasmic reticulum	Cobalamin transporter	Methylmalonic aciduria and homocystinuria, cblJ type, inborn error of vitamin B12 metabolism	mRNA [39,45,46], Protein [40]
ABCE1	OABP, RNS4I	Cytoplasm, mitochondria	Oligoadenylate binding protein, Translation		mRNA [39,40,45], Protein [41]
ABCF1	ABC50	Ribosome, nucleus, cytoplasm	Transcription, translation, innate immune responses		mRNA [39,40,45], Protein [41,48]
ABCF2					mRNA [39,40,45], Protein [41]
ABCF3					mRNA [39,40,46]
ABCG2	ABCP, MXR, BCRP	Mitochondria, Plasma membrane	Multidrug resistance,	Junior blood group system, gout	mRNA [39], Protein [49,50]
